# Data on the environmental exposure to lead in Iran

**DOI:** 10.1016/j.dib.2018.08.052

**Published:** 2018-08-28

**Authors:** Norouz Mahmoudi, Ali Mohammad Latifi, Mohammad Ali Amani, Hossein Masoumbeigi, Ghader Ghanizadeh

**Affiliations:** a Department of Environmental Health Engineering, School of Public Health, Iran University of Medical Sciences, Tehran, Iran; b Health Research Center, Life style Institute, Baqiyatallah University of Medical Sciences, Tehran; c Applied Biotechnology Research Center, Baqiyatallah University of Medical Sciences, Tehran, Iran

**Keywords:** Environmental exposure, Heavy metals, Lead, Blood lead level

## Abstract

The data was obtained to present the environmental and occupational exposure to lead in Iranian populations based on the published articles. To acquire the data, online resources including Google Scholar, Magiran, SID, Iranmedex, PubMed, and Science Direct were searched and 104 articles were found out of which 70 that focused on the level of lead in blood, urine, milk, and hair of different Iranian populations were selected. Since the results of the studies were not homogenous, it was not possible to carry out a meta-analysis. The average blood lead level (BLL) among workers, ordinary people, patients with specific diseases, addicts, and pregnant women, women in labor, infants, and children are presented in this article. The average BLL was compared to the standards.

**Specifications Table**

**Table:** 

Subject area	Environmental Health
More specific subject area	Public Health
Type of data	Tables
How data was acquired	The data was collected from different databases including Google Scholar, Magiran, SID, Iranmedex, PubMed, and Science Direct. All articles published by March 20, 2014 were included.
Data format	Raw and analyzed
Experimental factors	Based on their type and exposure intensity, the studies were classified into five groups: 1) workers and ordinary people, 2) patients with specific diseases, 3) addicts, 4) pregnant women and women in labor, and 5) infants and children.
Experimental features	Out of the 104 articles, 70 that were referable were used.
Data source location	Tehran, Tehran province, Iran.
Data accessibility	Data are included in this article

**Value of the data**

• The data provides information on the level of lead exposure among different Iranian groups, and is important for scientific community.
• The data clarifies protective, managerial, and policy-making measures of the risks involved with lead exposure more than before.
• The data can be useful as it collects all the available information about the blood lead level amount Iranians.

## 1 Data

Table 1, Table 2, Table 3, Table 4, Table 5 present the data of the studies in different populations. This data is extracted from 70 articles [1], [2], [3], [4], [5], [6], [7], [8], [9], [10], [11], [12], [13], [14], [15], [16], [17], [18], [19], [20], [21], [22], [23], [24], [25], [26], [27], [28], [29], [30], [31], [32], [33], [34], [35], [36], [37], [38], [39], [40], [41], [42], [43], [44], [45], [46], [47], [48], [49], [50], [51], [52], [53], [54], [55], [56], [57], [58], [59], [60], [61], [62], [63], [64], [65], [66], [67], [68], [69], [70]. As indicated in the tables, the level of the measured lead is different in different groups, with a higher level belonging to the addicts. The concentration levels of lead in blood were expressed in micromole per liter (µmol/l), which was turned into microgram per deciliter (µg/dl) by multiplying them by the constant of 20.72 (Table 2, Table 3, Table 4, Table 5).

**Table 1:** Studies related to mean blood lead concentration among adults (workers and ordinary people) in Iran.

**#**	**Study location**	**Sample environment**	**Sample Size**	**Lead level**	**References**
1	Kermanshah	Blood	150 workers of Kermanshah oil refinery	35.30±6.68 µg/dl	[1]
			70 workers of a textile factory near the refinery	19.7±3.91 µg/dl	
2	Tehran	Blood	497 workers of a battery recycling plant	43.31±17.95 µg/dl	[2]
3	Isfahan	Blood	142 workers of battery manufacturing plant	7.59±2.75 µg/dl	[3]
4	Isfahan	Blood	70 workers of battery industry with occupational exposure to lead	36.54±4.34 µg/dl	[4]
				8.82±3.96 µg/dl	
			76 office workers of the same factory (control)		
5	Tehran	Blood	60 soldering workers of an automotive company (experimental group)	36.3±9.9 µg/dl	[5]
				13.6±6.1 µg/dl	
			60 office workers of the same company (control group)		
6	Arak	Blood	67 Emarat lead and zinc mine and workers	9.64±3.281 µg/dl	[6]
			67 farmers near Emarat lead and zinc mine	5.07±3.061 µg/dl	
7	----	Blood	A 23-year-old worker of a lead battery recycling plant	130.53 µg/dl	[7]
8	Mashhad	Blood	105 workers of a battery manufacturing factory	32.2±13.7 µg/dl	[8]
9	Zanjan	Blood	40 workers of zinc smelting factory	16.06 µg/dl	[9]
				10.47 µg/dl	
			40 healthy men in the same area (control)		
10	Yazd	Blood	490 workers of Koushk lead and zinc mine	48.98±23.25 µg/dl	[10]
11	Tehran	Blood	32 welding workers in automotive industry	62 µg/dl	[11]
12	Yazd	Blood	70 workers of Koushk lead mine	7.06±4.84 µg/dl	[12]
				4.97±1.70 µg/dl	
			70 workers of Yazd Baf textile factory		
13	Tehran	Blood	11 welding workers in an automotive factory (control)	62.6±13.4 µg/dl	[13]
				67.2±12.8 µg/dl	
			8 welding workers in an automotive factory (experimental)		
14	Mashhad	Blood	108 workers of Mashhad traditional tile factories (2004)	52.05±32.32 µg/dl	[14]
				36.15±17.69 µg/dl	
			108 workers of Mashhad traditional tile factories (2005)		
15	Tehran	Blood	31 non-smoking workers	34.8±12.9 µg/dl	[15]
16	Tehran	Blood	50 battery manufacturing workers	96.7±27.9 µg/dl	[16]
17	Yazd	Blood	66 workers in different jobs (all individuals)	45.51±1.71 µg/dl	[17]
			21 battery repairmen	46.77±2.14 µg/dl	
			12 smoothers and painters	47.84±2.64 µg/dl	
			12 radiator and exhaust welders	59.42±3.87 µg/dl	
				36.14±2.76 µg/dl	
			15 workers directly involved with printing	32.17±6.84 µg/dl	
			6 workers indirectly involved with printing		
18	----	Blood	50 building painters	27.76±3.31 µg/dl	[18]
			54 individuals as the control group	11.81±4.35 µg/dl	
19	Hamadan	Blood	44 workers in gas stations	30.05±7.01 µg/dl	[19]
			44 individuals as the control group	17.31±3.46 µg/dl	
20	Zanjan	Hair	25 workers of lead ingot industry	131.7±93.4 µg/dl	[20]
				21.1±13.2 µg/dl	
			25 office workers of the same industry	27.9±14.1 µg/dl	
			25 citizens		
21	Isfahan	Urine	60 workers of gas stations	6.975±1.452 µg/dl	[21]
22	Naeein	Hair	25 workers of Nakhlak lead mine	43.52±27.72 µg/dl	[22]
			26 people living in surrounding villages (control)	38.17±43.3 µg/dl	
23	Tehran	Blood	15 workers of battery manufacturing industry (control)	63.3±3.4 µg Hb	[23]
				57.9±6.2µg Hb	
			15 workers of battery manufacturing industry (2^nd^ group)	59.6±4.9 µg Hb	
			15 workers of battery manufacturing industry (3^rd^ group)	50.9±5.7 µg Hb	
			15 workers of battery manufacturing industry (4^th^ group)		
24	Tehran	Blood	228 traffic policemen in Tehran	29.52±7.78 µg/dl	[24]
			68 police office employees	21.74±5.63 µg/dl	
25	Tehran	Urine	35 municipal workers	64.4±35.4 µg/dl	[25]
			35 control participants	9.2±3.2 µg/dl	
26	Tehran	Blood	40 male patients	100.32±18.42 µg/dl	[26]
			62 control participants	9.33±18.42 µg/dl	
27	Tehran	Blood	49 female patients	27.4±3.10 µg/dl	[27]
			51 control women	12.6±2.30 µg/dl	
28	Tehran	Blood	41 male patients	110.3±37.5 µg/dl	[28]
29	Arak	Blood	1,142 citizens of Arak	13.42 µg/dl	[29]
30	Ravar, Feyz Abad	Blood	30 men living around a lead mine	22 µg/dl	[30]
				17 µg/dl	
			30 men elsewhere (control)		
31	Babol	Blood	427 infected with lead	110.2 µg/dl	[31]
			430 healthy individuals (control)	14.08 µg/dl	
32	Tehran	Blood	100 guidance male students	11.63 µg/dl	[32]
				7.21 µg/dl	
			100 guidance female students

**Table 2:** Studies related to mean blood lead concentration in Iranian patients with specific diseases.

**#**	**Study location**	**Environment sample**	**Sample size**	**Lead level**	**References**
1	Sari	Blood	75 patients with asthma	4.98±3.11 µg/dl	[33]
			65 healthy individuals (control)		
				3.35±1.64 µg/dl	
2	Tehran	Blood	93 hemodialysis patient	9.7±3.7 µg/dl	[34]
3	Ahwaz	Blood	33 dialysis patients	2.714±0.64 µg/dl	[35]
			33 control participants		
				1.67±0.68 µg/dl	
4	Sari	Blood plasma	32 esophageal cancer patients	52±15 µg/dl	[36]
				56±8 µg/dl	
			32 control individuals		
5	Tehran	Blood	80 patients with blood pressure	5.1±0.4 µg/dl	[37]
				2.7±0.3 µg/dl	
			80 healthy individuals as the control group

**Table 3:** Studies related to mean blood lead concentration among drug users in Iran.

**#**	**Study location**	**Environment sample**	**Sample size**	**Lead level**	**References**
1	Mashhad	Blood	1 oral addict	196.1 µg/dl	[38]
2	Tehran	Blood	39 addicts	57.04±46.03 µg/dl	[39]
			39 control participants	16.7±12.51 µg/dl	
3	Tehran	Blood	7 lead-poisoned addicts in Loghman-e-Hakim Hospital	109±37.6 µg/dl	[40]
4	Tehran	Blood	One 27-year-old addict worker	154 µg/dl	[41]
			One 68-year-old addict worker	180 µg/dl	
5	Rafsanjan	Blood	22 addicts	21.9±13.24 µg/dl	[42]
				8.6±3.5 µg/dl	
			22 control participants		
6	Yazd	Blood	1 oral addict (a 46-year-old man, copper smelting worker)	90 µg/dl	[43]
7	Kerman	Blood plasma	50 opium addicts	329.94±14.76 µg/dl	[44]
			43 non-addicts as control group	353.27±114.15 µg/dl	
8	Tehran	Blood	One 25-year-old addict	350 µg/dl	[45]
9	Tehran	Blood	One 52-year-old oral addict	116 µg/dl	[46]
10	Tehran	Blood	One 41-year-old addict	118 µg/dl	[47]
11	Tehran	Blood	61 male addicts living in Tehran	13.811±6.543 µg/dl	[48]
				10.184±5.138 µg/dl	
			40 female addicts living in Tehran	12.375±5.642 µg/dl	
			All male and female addicts		
12	Hamadan	Blood	Lead-poisoned patient 1 (man, 43 years old)	99 µg/dl	[49]
				77 µg/dl	
				104 µg/dl	
			Lead-poisoned patient 2 (man, 25 years old)		
			Lead-poisoned patient 3 (man, 23 years old)		
13	Tehran	Blood	A 34-year-old addict	95 µg/dl	[50]
				81 µg/dl	
			A 57-year-old addict		
			A 45-year-old addict	37.5 µg/dl	
14	Tehran	Blood	A 40-year-old addict	Over 200 µg/dl	[51]
15	Tehran	Urine	Chronic lead poisoning in a 45-year-old male addict	244 µg/dl	[52]

**Table 4:** Studies focused on mean blood lead level among pregnant women, women in labor, and infants in Iran.

**#**	**Study location**	**Environment sample**	**Sample size**	**Lead level**	**References**
1	Tehran	Blood	961 pregnant women with timely deliver	4.7±4.9 µg/dl	[53]
			72 pregnant women with premature delivery	4.8±4.6 µg/dl	
2	Tehran	Blood	75 women (mother’s blood wile delivery)	2.73±0.94 µg/dl	[54]
				2.83±1.31 µg/dl	
			75 neonates of the same mothers (umbilical cord blood)		
3	Tehran	Blood	348 singleton pregnant women aging 16-32 (the first 3 months of pregnancy)	3.8±2 µg/dl	[55]
4	Tehran	Blood	232 women in labor (total)	3.8±2 µg/dl	[56]
				4.61±2.37 µg/dl	
				3.69±1.85 µg/dl	
			36 women in labor with PROM		
			269 women in labor with non-PROM		
5	Zarrin Shahr, Isfahan	Breast milk	27 mothers	4.6 µg/dl	[57]
6	Mashhad	Blood	40 mothers with a neonate weighing below 2500 gr	10.49±26.4 µg/dl	[58]
				12.46±17.5 µg/dl	
			40 mothers with a neonate weighing over 2500 gr		
7	Ardabil	Blood	65 mothers with a infants weighing below 2500 gr	Below 1 µg/dl	[59]
				Below 1 µg/dl	
			65 mothers with a neonate weighing over 2500 gr		
8	Isfahan	Blood	32 mothers with intrauterine growth retard (IUGR)	12.465±1.91 µg/dl	[60]
				10.747±1.675 µg/dl	
			32 neonates with intrauterine growth retard (IUGR)		
				13.562±2.691 µg/dl	
				11.308±1.908 µg/dl	
			34 mothers		
			34 neonates		
9	Tehran-Rasht	Blood	86 mothers in a non-polluted region	7.6±41 µg/dl	[61]
				5.9±3.2 µg/dl	
				9.07±8.41 µg/dl	
				6.6±5.18 µg/dl	
			86 infants in a non-polluted region		
			85 mothers in a polluted region		
			85 infants in a polluted region		
10	Tehran	Blood	31 preeclampsia	5.09±2.01 µg/dl	[62]
				4.82±2.22 µg/dl	
			465 control participants		
11	Tehran	Blood	55 pregnant women with high blood pressure	5.7±2 µg/dl	[63]
				4.8±1.9 µg/dl	
			55 pregnant women with normal blood pressure (control)

**Table 5:** Studies focusing on blood lead concentration among Iranian children.

**#**	**Study location**	**Environment sample**	**Sample size**	**Lead level**	**References**
1	Guilan	Blood plasma	90 ill children	11.643 µg/dl	[64]
			90 healthy children	4.924 µg/dl	
2	Birjand	Teeth	108 children aging 5-12 years (deciduous teeth)	1.96±1.62 µg/dl	[65]
3	Tehran	Blood	100 children with hyperactivity and attention deficit	7.2±2.365 µg/dl	[66]
				7.186±3.186 µg/dl	
			100 healthy children		
4	Mashhad	Blood	32 children aging 3-7 years old	16.381±5.719 µg/dl	[67]
5	Zanjan	Blood	45 children aging 7-11 living around Anguran lead mine	36.7±24.67 µg/dl	[68]
				15.57±13.35 µg/dl	
			36 children aging 7-11 (control)		
6	Mashhad	Blood	206 children aging 1-7 years	12.195±3.359 µg/dl	[69]
7	Semnan	Blood	320 primary students aging 6-11 in Semnan’s schools	21% below 10 µg/dl	[70]
				74% between 10 and 20 µg/dl	
				5% over 20 µg/dl	

## 2 Experimental design, materials and methods

The data is based on the articles that were sporadically carried out on certain groups and different cities and published in domestic and foreign journals. The articles were selected from different databases including Google Scholar, Magiran, SID, Iranmedex, PubMed, and Science Direct. While searching the articles, keywords like lead, occupational exposure to lead, lead measuring, human lead contamination, BLL, blood lead level, lead poisoning, lead toxicity, lead exposure were used and their Persian equivalents in Persian websites. All articles published by March 20, 2014 were included. First, all articles on lead exposure carried out in Iran were collected. At this stage, all articles that contained the mentioned keywords in their title or abstract were included in the primary list. Afterwards, a checklist of necessary study information, including study location, study year, sample environment, sample size, and average blood lead level (BLL), was prepared for final evaluation. Searching and extracting of the data was independently carried out by one person. A total of 104 articles that were available by March 20, 2014 were examined. Out of the 104 articles, 70 that were referable were taken used [1], [2], [3], [4], [5], [6], [7], [8], [9], [10], [11], [12], [13], [14], [15], [16], [17], [18], [19], [20], [21], [22], [23], [24], [25], [26], [27], [28], [29], [30], [31], [32], [33], [34], [35], [36], [37], [38], [39], [40], [41], [42], [43], [44], [45], [46], [47], [48], [49], [50], [51], [52], [53], [54], [55], [56], [57], [58], [59], [60], [61], [62], [63], [64], [65], [66], [67], [68], [69], [70]. Due to the heterogeneity of the collected data, it was impossible to carry out a meta-analysis. Based on their type and exposure intensity, the studies were classified into five groups: 1) workers and ordinary people, 2) patients with specific diseases, 3) addicts, 4) pregnant women and women in labor, and 5) infants and children.

workers, ordinary people, patients with specific diseases, addicts, and pregnant women, women in labor, infants, and children.
